# Perennial growth of hermatypic corals at Rottnest Island, Western Australia (32°S)

**DOI:** 10.7717/peerj.781

**Published:** 2015-02-24

**Authors:** Claire L. Ross, James L. Falter, Verena Schoepf, Malcolm T. McCulloch

**Affiliations:** 1 UWA School of Earth and Environment and Oceans Institute, University of Western Australia, Crawley, Western Australia, Australia; 2 Australian Research Council Centre of Excellence for Coral Reef Studies, University of Western Australia, Crawley, Western Australia, Australia

**Keywords:** Coral, Calcification, Extension rates, Seasonality, High latitude, Coral bleaching, Western Australia

## Abstract

To assess the viability of high latitude environments as coral refugia, we report measurements of seasonal changes in seawater parameters (temperature, light, and carbonate chemistry) together with calcification rates for two coral species, *Acropora yongei* and *Pocillopora damicornis* from the southernmost geographical limit of these species at Salmon Bay, Rottnest Island (32°S) in Western Australia. Changes in buoyant weight were normalised to colony surface areas as determined from both X-ray computed tomography and geometric estimation. Extension rates for *A. yongei* averaged 51 ± 4 mm y^−1^ and were comparable to rates reported for Acroporid coral at other tropical and high latitude locations. Mean rates of calcification for both *A. yongei* and *P. damicornis* in winter were comparable to both the preceding and following summers despite a mean seasonal temperature range of ∼6 °C (18.2°–24.3 °C) and more than two-fold changes in the intensity of downwelling light. Seasonal calcification rates for *A. yongei* (1.31–2.02 mg CaCO_3_ cm^−2^ d^−1^) and *P. damicornis* (0.34–0.90 mg CaCO_3_ cm^−2^ d^−1^) at Salmon Bay, Rottnest Island were comparable to rates from similar taxa in more tropical environments; however, they appeared to decline sharply once summer temperatures exceeded 23 °C. A coral bleaching event observed in December 2013 provided further evidence of how coral at Rottnest Island are still vulnerable to the deleterious effects of episodic warming despite its high latitude location. Thus, while corals at Rottnest Island can sustain robust year-round rates of coral growth, even over cool winter temperatures of 18°–19 °C, there may be limits on the extent that such environments can provide refuge against the longer term impacts of anthropogenic climate change.

## Introduction

Declining rates of coral growth have stimulated debate over the future of coral reefs and the mechanisms or strategies that may be available for hermatypic corals to endure in the face of Earth’s rapidly changing climate. Recent studies have suggested that range expansions to ‘high latitude refugia’ (i.e., above 28°N and 28°S) could offer one such pathway for survival from increasing seawater temperatures (Denis et al., 2013; Couce, Ridgwell & Hendy, 2013). Already there is evidence of various coral species actively expanding their distributions polewards (Yamano, Sugihara & Nomura, 2011; Baird, Sommer & Madin, 2012), particularly to regions where there are fossilized reefs signifying the past suitability of those areas to support extensive coral growth during previous warmer geological periods (McCulloch & Esat, 2000; Greenstein & Pandolfi, 2008). The suitability of high latitude environments as future refugia for coral reefs, however, remains unclear at present (Pandolfi et al., 2011; Van Hooidonk et al., 2014) given the recent occurrence of high latitude coral bleaching events (Harrison, Dalton & Carroll, 2011; Smale & Wernberg, 2012). Thus, the capacity for corals to persist under future global climate change may also largely depend on their capacity to cope with, or acclimatize to, changing environmental conditions (McCulloch et al., 2012; Rodolfo-Metalpa et al., 2014; Keshavmurthy et al., 2014).

Temperature is generally thought to be the primary environmental driver controlling the geographic distribution of coral, rates of coral calcification and reef formation worldwide (Veron, 1995; Lough & Barnes, 2000; Marshall & Clode, 2004); however, decoupling the effects of seasonal changes in light from seasonal changes in temperature is problematic due to their close phase coherence (Falter et al., 2012; Venti, Andersson & Langdon, 2014). Studies have shown that the growth rates of tropical coral increase with temperature until an optimum is reached, usually between 25 °C and 28 °C, or 2°–3 °C below the mean monthly maximum (Jokiel & Coles, 1977; Marshall & Clode, 2004). Provided the coral is not under some form of stress, any elevation in temperature should increase metabolism and result in faster rates of biomineralization (Burton & Walter, 1987; McCulloch et al., 2012), while higher light levels increase skeletal growth through the increased production of photosynthetically fixed carbon by the zooxanthellae symbiont (Gattuso, Allemand & Frankignoulle, 1999). Thus, the lower and more seasonally variable temperature and light levels characteristic of high latitude environments generally result in lower and more seasonally dependent rates of calcification (Crossland, 1981; Rodolfo-Metalpa et al., 2009). Consequently, the extensive stands of scleractinian corals needed to build and sustain reef structures are mostly confined to tropical latitudes (i.e., between 22°N and 22°S) where light levels and aragonite saturation states are high (light levels >22 mol m^−2^ d^−1^ and Ω_ar_ > 3.5; Kleypas, McManus & Menez, 1999), nutrient concentrations are generally low (<1 µM; Atkinson & Falter, 2003), and water temperatures are maintained above 18 °C (Veron, 1995). Subsequently, while there are a few examples of well-developed living coral reef structures at high latitude (e.g., Bermuda in the North Atlantic at 32.3°N, Lord Howe Island in Australia at 31.3 °S, and Iki Island in Japan at 33.5°N), many high latitude coral communities do not form massive coral reef structures (Veron, 1995).

**Figure 1:**
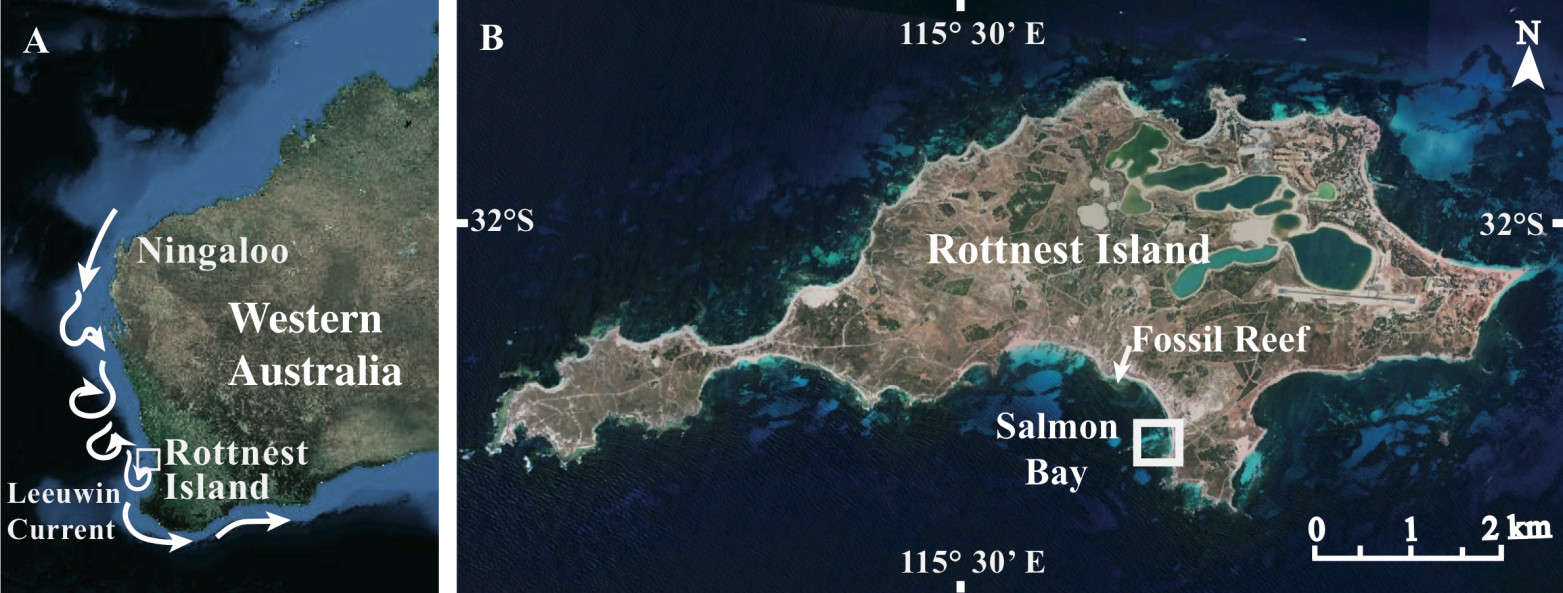
Site maps. (A) Map of Western Australia showing Rottnest Island in relation to the Leeuwin Current, and (B) close-up map of Rottnest Island. Map image: Google Earth, Digital Globe, NASA.

Rottnest Island (32°S, 115°E) off the coast of Perth in Western Australia (WA) hosts a range of temperate to sub-tropical habitats due to the influence of the perennial Leeuwin Current, which transports warm, oligotrophic water poleward along the WA coastline (Fig. 1A). These waters support a unique combination of tropical and temperate marine fauna including over 25 scleractinian coral species (Veron & Marsh, 1988). Although Pocilloporid coral have long colonized the shallow benthos surrounding Rottnest Island (Veron & Marsh, 1988), the distribution of Acroporid coral at Rottnest Island remains limited to a small embayment on the south side (Fig. 1B). However, based on our estimate from baseline surveys, the number of living colonies has increased roughly 100-fold since its initial distribution, which was reported by Marsh (1992) over 20 years ago (2 colonies first recorded in 1988 vs. 218 colonies in the present study; Fig. S1). Thus, Rottnest Island appears to be representative of a high latitude habitat in the early stages of colonization and expansion by hermatypic coral more commonly found in sub-tropical and tropical waters. Indeed, fossil records indicate that extensive Acroporid-dominated reefs existed at Rottnest Island 1 km to the west of the modern *Acropora* spp. coral community during the Last Interglacial (∼125 ka ago) when ocean temperatures were 1° to 2 °C higher than present day (Szabo, 1979). To date, however, only two studies have measured seasonal changes in colony extension rates (mm y^−1^) in two of the coral species at Rottnest Island (*Acropora yongei* and *Pocillopora damicornis*; Marsh, 1992; Ward, 1995). To our knowledge there have been no measurements of surface area-normalized skeletal growth or calcification at this site (mg CaCO_3_ cm^−2^ d^−1^), nor any corresponding measurements of the environmental conditions under which these coral have been growing. Thus, we do not know the degree to which these coral species have acclimatized to the conditions at Rottnest Island or to what extent this habitat can support the continued growth and accumulation of hermatypic coral over the long term.

The present study was aimed at measuring how calcification rates of *A. yongei* and *P. damicornis* at Rottnest Island respond to seasonally varying changes in light, temperature and seawater chemistry over two summers and one winter. We then compare our results to the seasonal growth patterns of similar coral taxa living in other high and low latitude reef environments to better determine the suitability of this high latitude environment as coral refugia, thereby supporting the continued growth of hermatypic coral given anticipated trends in climate-driven ocean warming. This study will provide important baseline calcification rate data for high latitude hermatypic branching corals at the edge of their geographical distributions in WA. We initially hypothesized (1) that both *A. yongei* and *P. damicornis* at Rottnest Island would exhibit a growth ‘hiatus’ or decrease in rates of calcification during the winter months (Fallon et al., 1999; Burgess et al., 2009), and (2) that calcification rates for both species would be lower than those of similar coral taxa living in lower latitude reef environments (Harriott, 1999; Foster et al., 2014); both as a result of the lower and more seasonally variable light levels and seawater temperatures typical of high latitude environments. Instead, we show not only that calcification rates in *A. yongei* and *P. damicornis* exhibited minimal coherent seasonality, but that each of these species calcified at rates comparable to the same or similar species growing in more tropical habitats. We further found, however, that coral living in this high latitude environment were still prone to bleaching, likely as a result of episodic thermal stress.

## Materials and Methods

### Ethics statement

The Rottnest Island Authority (RIA) provided approval and permits for this research (#2012/164683 and 2014/227143). The Government of Western Australia Department of Parks and Wildlife (DPaW) provided research permits and license to take fauna for scientific purposes (#SF009096 and #SF009705) and the Government of Western Australia Department of Fisheries provided research permits and exemption from the Fish Resources Management Act 1994 (#2202 and #2410).

### Site description

This study was conducted at Rottnest Island, located approximately 20 km offshore Perth, WA (32°S, 115°E, Fig. 1B). We focused on two locations within the far eastern end of Salmon Bay given that this is the only known location at Rottnest Island where *A. yongei* coral are currently reported to occur (*P. damicornis* coral can be found at multiple sites). Coral and filamentous turf algae co-inhabit this inner reef environment exhibiting mixed assemblages typical of other near-shore habitats at high-latitude (Banks & Harriott, 1995; Thomson & Frisch, 2010). Rottnest Island can be subject to strong southwesterly swells and storms with wave heights in excess of 2 m (Richardson, Mathews & Heap, 2005); however, our study site within Salmon Bay is protected from the south-westerly swell by a limestone reef platform located ∼300 m seaward from shore (Marsh, 1992).

### Environmental conditions

Continuous measurements of seawater temperature in Salmon Bay, Rottnest Island were made using HOBO temperature loggers (±0.2 °C, Onset Computer Corp.). Daily down-welling planar photo-synthetically active radiation (PAR in mol m^−2^ d^−1^) for Rottnest Island was measured from December 2013 through July of 2014 using light loggers (±5%, Odyssey Data Recording) calibrated using a high precision LiCor 192A cosine PAR sensor that was factory calibrated. Additional in-water measurements of light were made on the 24th January 2014 and on the 30th July 2014 to estimate depth-dependent light attenuation coefficients (*k_d_*) in summer and winter, respectively. During each field sampling trip, discrete samples of water were collected from the study site using a 2.2-L Van Dorn bottle and measurements of pH on the total hydrogen ion concentration scale (pH_T_) were made using a Schott Handylab pH 12 m (±0.03) using both seawater (‘Tris’) and NBS buffers and calibrated against a certified reference pH buffer provided by Andrew Dickson at the Scripps Institute of Oceanography (Batch #123). A SeaFET ocean pH sensor (Satlantic, Halifax, Nova Scotia, Canada) was also deployed at a depth of 2.5 m in Salmon Bay for 5 days in January 2014 (i.e., representing summer) and 12 days in July 2014 (i.e., representing winter) to measure diurnal changes in pH_T_ (±0.03). Additional seawater samples were collected and filtered with 0.7-µm nominal glass fibre filters (Whatman GF/F; Sigma Aldrich, St. Louis, Missouri, USA) and taken back to UWA for the analysis of total alkalinity (TA) and dissolved inorganic nutrients. TA (±5 µeq kg^−1^) was measured using a modified version of the spectrophotometric approach developed by Yao & Byrne (1998). Dissolved Inorganic Carbon (DIC), the partial pressure of dissolved carbon dioxide (*p*CO_2_), and aragonite saturation state (Ω_ar_) were calculated from measured pH_T_, TA, and temperature using the CO2SYS program (Lewis, Wallace & Allison, 1998) and assuming seasonally dependent salinity of between 35.4 and 35.8 (Lourey, Dunn & Waring, 2006). Concentrations of dissolved ammonium (±0.1 µM), nitrate + nitrite (±0.05 µM), and phosphate (±0.03 µM) were measured using a QuikChem 8500 Series 2 Flow Injection Analysis (FIA) System (Lachat Instrument, Milwaukee, Wisconsin, USA).

### Coral calcification rates

**Figure 2:**
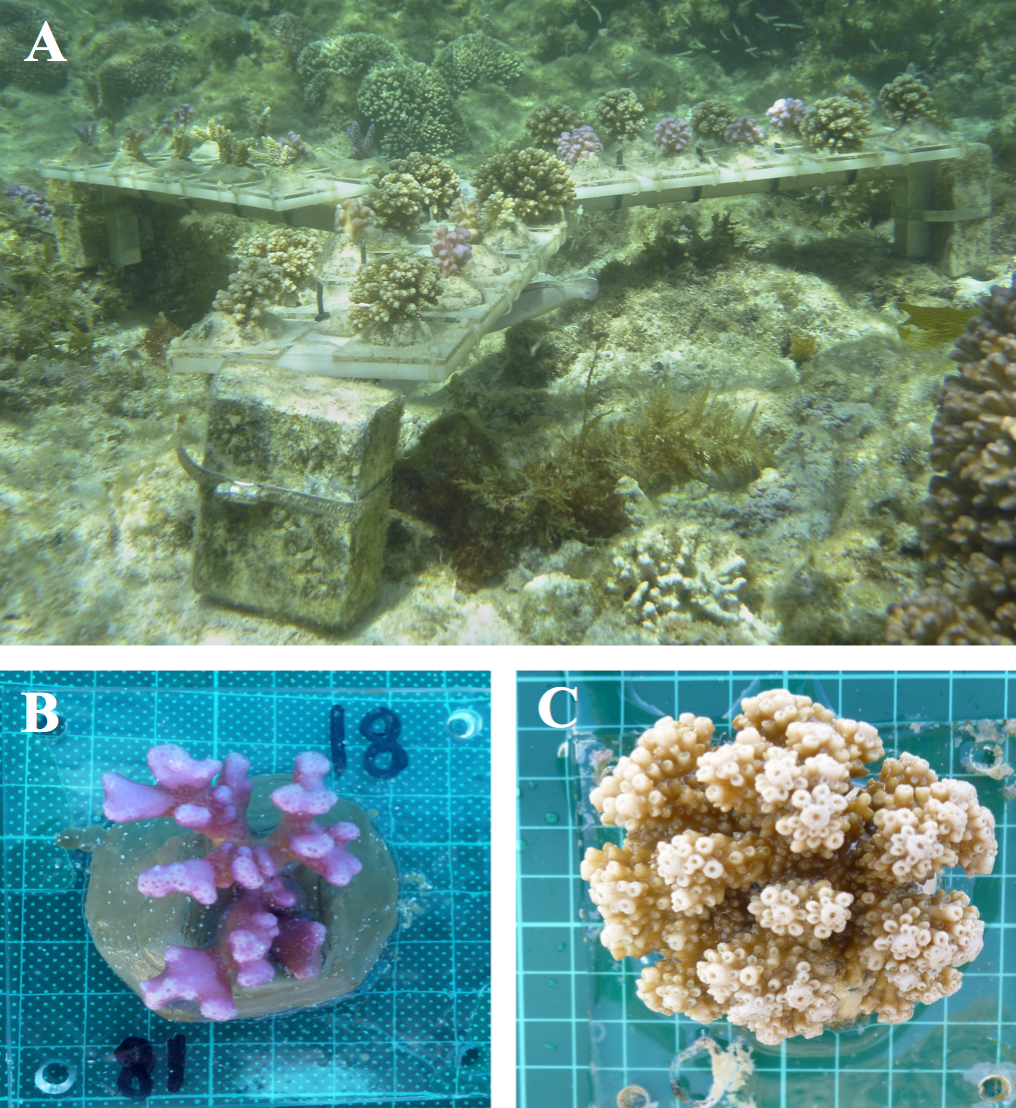
Sample coral colonies growing on tiles. Photographs of (A) the aluminium tripod and the coral species (B) *Pocillopora damicornis,* and (C) *Acropora yongei* growing on the acrylic tiles. Photographs by C. Ross (2013).

Branches of *A. yongei* and *P. damicornis* were collected from replicate colonies (*n* = 10 for *A. yongei*, *n* = 9 for *P. damicornis*; one branch from each replicate colony) within Salmon Bay, Rottnest Island in December 2012. Samples were buoyantly weighed, epoxied onto acrylic plates (Fig. 2; Z-Spar™ Splash Zone Compound A-788) and then buoyantly re-weighed to determine the weight of the tile and marine epoxy. Each sample tile was then secured randomly onto an aluminium tripod (Fig. 2) or a concrete block deployed at two different locations within Salmon Bay (depths of 1.5 m and 2.5 m, respectively), close to the parent colonies. Tiled coral samples were first allowed to recover for ∼4 weeks in situ before their buoyant weights were initially measured in January 2013. Some samples died at the very beginning of the study (*n* = 5 of *A. yongei* and *n* = 3 of *P. damicornis*) and could not be included in the study, thus resulting in a final sample size of *n* = 5 for *A. yongei* and *n* = 6 for *P. damicornis* at the beginning of the experiment (i.e., January–February 2013). To increase our overall sample number, additional coral samples were deployed in February 2013 (*n* = 11 of *A. yongei* and *n* = 3 of *P. damicornis*) all of which survived, giving a total sample size of *n* = 16 for *A. yongei* and *n* = 9 for *P. damicornis*. In November 2013, we deployed even more samples of each coral species to investigate potential size effects on rates of calcification for both species (*n* = 6 of *A. yongei* and *n* = 4 of *P. damicornis*); however, data from these colonies were used solely to examine relationships between calcification and colony size (Figs. 7C and 7D) and thus were not included in the analysis of seasonal growth trends.

At 1–3 monthly intervals, coral samples on tiles were detached from their support structures, transported to the shore on snorkel, weighed on shore using the buoyant weight technique (Bak, 1973; Jokiel, Maragos & Franzisket, 1978; Davies, 1989), and then returned to the water ∼2 h later. On shore, the coral colonies were kept in aerated 50-L containers and the plastic tiles were cleared of any additional growth of epilithic organisms before being weighed. The deployment of two control tiles consisting solely of a tile with epoxy and no coral colony indicated that the long-term changes in the mass of the tiles due to residual epiphytic growth after cleaning were less than 5% of the measured changes in buoyant weight over any given growth interval.

Changes in skeletal mass were calculated from the change in buoyant weight normalized to total bioactive surface area measured using both geometric estimation (Naumann et al., 2009) and X-ray computed tomography (X-ray CT; Laforsch et al., 2008). For the geometric estimation, colony surface area was calculated by summing the total area of all branches assuming cylindrical branch geometry. For the more accurate X-ray CT, 3D scans of colonies were created using the SkyScan *In-vivo* micro X-ray CT (UWA Centre for Microscopy, Characterization and Analysis) calibrated against two different rectanguloid sections of coral skeleton. Reconstructions of the scans were performed by NRecon software and surface area analyses were performed in CTAn software (Bruker Microct, Kontich, Belgium). To prevent sacrificing any living sample colonies growing on tiles during the course of the experiment, the surface area of each colony was calculated using regressions of total surface area versus dry skeletal weight created from a range of separately collected colonies of *P. damicornis* and *A. yongei* whose individual masses spanned a very large range from 2–370 g (*A. yongei*: RMSE = 1.1 cm^2^, *R*^2^ = 0.99, *y* = 4.1*x*^0.97^, *n* = 26; *P. damicornis*: RMSE = 1.0 cm^2^, *y* = 2.1*x*^1.20^, *R*^2^ = 0.99, *n* = 14). Due to size restraints, the X-ray CT scanner was only used to analyze small colonies less than 30 g (<15 cm); therefore, for larger colonies, we estimated total colony surface area using geometric estimation and then corrected these estimates based on regressions of X-ray CT surface area against geometric estimation surface area (*A. yongei*: *y* = 1.20*x*–7.64, RMSE = 3.8 cm^2^, *R*^2^ = 0.98, *n* = 10 and *P. damicornis*: *y* = 1.19*x*–4.13, RMSE = 5.9 cm^2^, *R*^2^ = 0.95, *n* = 10). Rates of calcification for each coral colony were then calculated based on the change in dry skeletal mass, the total surface area estimated from the initial and final mass, and the number of days between buoyant weighing.

### Coral linear extension rates

We measured the linear extension rates of three coral branches within five different *A. yongei* colonies randomly selected from across the eastern part of Salmon Bay to assess how growth (extension) rates for *A. yongei* varied across the total study area. Each branch was tagged 2 cm from the growing tip using coloured plastic cable ties (Shinn, 1966; Anderson, Pratchett & Baird, 2012) and measured at 1–3 month intervals. Linear extension rates were not measured for *P. damicornis* because the highly reticulate morphology of this coral made distal growth measurements unreliable.

### Data analyses

Seasonal changes in TA, pH_T_, Ω_ar_ and nutrients were tested using an independent *t* test. Repeated measures analysis of variance (rANOVA) with a Bonferroni post-hoc test was performed to test for significant differences in rates of calcification and rates of linear extension across time. After finding no significant effect of colony location on linear extension rate (*F*_28,70_ = 1.4, *p* > 0.05), colony data were pooled. A priori analyses further indicated that rates of calcification at the two locations within Salmon Bay (i.e., tripod at 1.5 m and brick at 2.5 m) did not significantly differ (*F*_1,145_ = 1.5, *p* > 0.05); therefore, these data were also pooled.

**Figure 3:**
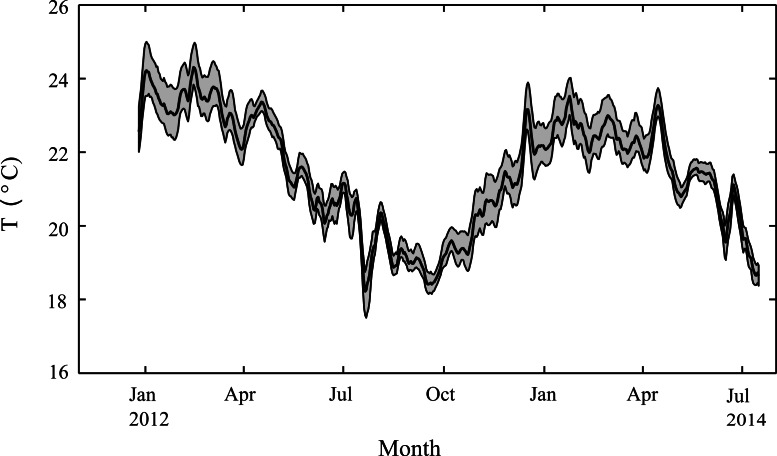
Seasonal changes in seawater temperature. Water column temperatures at Salmon Bay, Rottnest Island from December 2012 through July 2014, smoothed with a 1-week moving average. The heavy black line represents average daily temperatures while the grey regions represent the range between hourly minimum and maximum temperatures for each day.

We tested for differences between the samples that survived the initial deployment and those new samples that were deployed ∼8 weeks later in Feb 2013 using a one-way ANOVA. The study-averaged calcification rates for corals from the two deployments differed by less than 6% for both species (*A. yongei*: 1.67 vs. 1.60 mg CaCO_3_ cm^−2^ d^−1^; *P. damicornis*: 0.66 vs. 0.70 CaCO_3_ cm^−2^ d^−1^) and we found that there were no significant differences between the two deployments (*A. yongei*: *F*_1,146_ = 0.79, *p* > 0.05, *P. damicornis*: *F*_1,77_ = 0.31, *p* > 0.05); therefore, these data were pooled. Changes in seasonally averaged calcification rates and linear extension rates were also tested using a one-way ANOVA. For all data analyses, measurements from November through April were treated as ‘summer’ and measurements from May through October as ‘winter’ based on our temperature records (Fig. 3).

Statistical significance was determined at the *p* <0.05 level and dependent variables were tested for normality (Shapiro–Wilk test). Before conducting rANOVA tests, the dependent variables were tested for sphericity (Mauchly’s test). When calcification rate data for *A. yongei* and *P. damicornis* deviated from the assumptions of sphericity, we adjusted the degrees of freedom using the Greenhouse-Geisser adjustment with an epsilon of 0.35 and 0.43, respectively (Quinn & Keough, 2002). Before conducting linear regression analyses we tested for independence of observations (Durbin-Watson statistic) and homoscedasticity in the variance of errors, as assessed by inspection of the regression-standardized residuals and regression standardized predicted values. All statistical analyses were performed using SPSS version 22 statistical software (IBM, Foster City, California, USA) and time series analyses were performed using Matlab version 7.2 (The Mathworks, Natick, Massachusetts, USA).

## Results

### Environmental data

**Figure 4:**
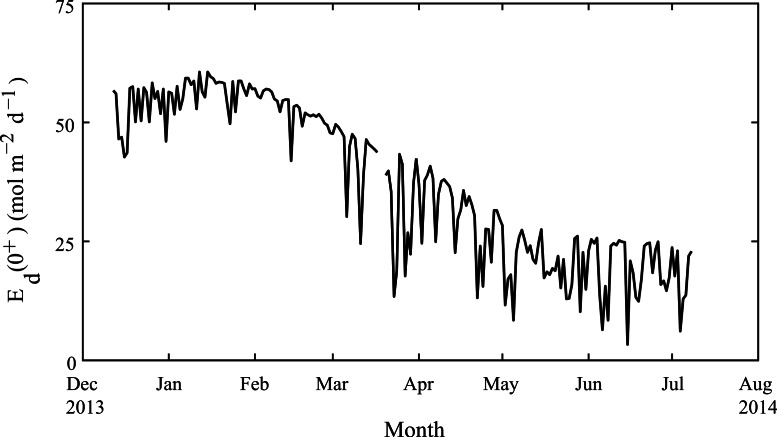
Seasonal changes in light. Daily-integrated down-welling planar PAR irradiance in mol m^−2^ d^−1^ measured in air from December 2013 through July 2014 at Rottnest Island.

Weekly average water temperatures were found to range from 18.2 °C (July 2013) to 24.3 °C (February 2013) giving a seasonal temperature range of ∼6 °C (Fig. 3). These findings are consistent with earlier measurements made at another shallow nearshore site between 1978 and 1991 less than 1 km from our study site (18.5°–23.5 °C; Marsh, 1992). The daily range in hourly mean temperatures (minimum to maximum) varied seasonally but were generally minor, ranging on average from 0.4 °C in July to 1.2 °C in January. Daily average light at Rottnest Island was, however, more than two-fold higher in summer (54 mol m^−2^ d^−1^) than in winter (19 mol m^−2^ d^−1^; Fig. 4). The diffuse attenuation coefficient for seawater (*k_d_*) in Salmon Bay measured at the depth of the tripod (1.5 m) was very low in both seasons (0.053 m^−1^ during summer vs. 0.075 m^−1^ during winter), corresponding to a seasonal range in light reaching the benthos of 15–48 mol m^−2^ d^−1^. Light levels were ∼80% of surface values at the tripod site (1.5 m) and ∼70% at the brick site (2.5 m). Thus, light levels these two deployment locations generally differed by only ∼10%.

TA did not differ between seasons (2,282 ± 18 in summer vs. 2,297 ± 4 in winter, *t* test, *t*(26) = − 1.19, *p* > 0.05, Table 1), and while daytime seawater pH_T_ ranged from 7.9–8.19, on average summer pH_T_ was only 0.03 pH units higher than winter (mean ± SE = 8.13 ± 0.02 vs. 8.10 ± 0.02, *t* test, *t*(33) = 1.04, *p* > 0.05, Table 1). Continuous measurements of water column pH_T_ over diurnal cycles indicate daily average values that were close to equilibrium with the atmosphere (8.03 in summer and 8.07 in winter vs. 8.047 and 8.048; *T* = 22.1 °C and 20.7 °C, TA = 2,301, and *p*CO_2_ = 395 µatm, Fig. S3). The higher daytime discretely measured pH was not unexpected given the observed diurnal pH amplitudes of around ±0.1 for both seasons and uncertainties in their measurement (7.9–8.15 ± 0.03 in summer and 8.0–8.2 ± 0.03 in winter, Fig. S3). The magnitude of these diurnal changes likely varied over time in accordance with changes in both offshore wave climate and local rates of community metabolism (Zhang et al., 2012; Falter et al., 2013). Consequently, Ω_ar_ was on average ∼0.6 lower in winter than in summer based on discrete daytime samples (3.28 ± 0.12 vs. 3.89 ± 0.14, respectively, *t* test, *t*(33) = 2.54, *p* < 0.05, Table 1), but only ∼0.4 lower in winter than in summer based on continuously measured pH averaged over many diurnal cycles (2.67 ± 0.15 vs. 3.08 ± 0.17). Ammonium concentrations were comparable across summer and winter (0.4–0.5 mmol m^−3^, *t* test, *t*(12) = 1.12, *p* > 0.05, Table 1), while nitrate concentrations were three-fold higher in winter than in summer (0.77 vs. 0.25 mmol m^−3^; *t* test, *t*(13) = − 6.22, *p* < 0.05, Table 1), and phosphate concentrations were six-fold higher in winter than in summer (0.19 vs. 0.03 mmol m^−3,^, *t*-test, *t*(13) = − 4.12, *p* < 0.05, Table 1).

**Table 1:** Measured abiotic environmental variables. Seasonal averages (mean ± SE) of light, temperature and biogeochemical data taken from continuous data loggers and from discrete daytime water quality measurements at Salmon Bay, Rottnest Island in summer (November–April) and winter (May–October) between 2011 and 2014.

Parameter	Unit	*n*	Summer	*n*	Winter
Light/*E_d_*(0^+^)	mol m^−2^ d^−1^	–	45.9 ± 0.6	–	19.3 ± 0.3
Temperature	°C	–	23.0 ± 0.2	–	19.5 ± 0.2
Total alkalinity	µmol kg^−1^	18	2,282 ± 18	10	2,297 ± 4
pH_T_	–	25	8.13 ± 0.02	10	8.10 ± 0.02
Ω_ar_	–	25	3.89 ± 0.14	10	3.28 ± 0.12
*p*CO_2_	µatm	25	310 ± 20	10	339 ± 17
Carbonate	µmol kg^−1^	25	248 ± 9	10	211 ± 8
DIC	µmol kg^−1^	25	1928 ± 20	10	1997 ± 14
Ammonium	mmol m^−3^	8	0.50 ± 0.03	6	0.43 ± 0.06
Nitrate	mmol m^−3^	9	0.25 ± 0.04	6	0.77 ± 0.08
Phosphate	mmol m^−3^	9	0.03 ± 0.01	6	0.19 ± 0.04

**Notes.**

pH_T_ pH on the total hydrogen ion concentration scale Ω_ar_ aragonite saturation state *p*CO_2_ partial pressure of dissolved carbon dioxide DIC dissolved inorganic carbon

### Rates of calcification and linear extension

**Figure 5:**
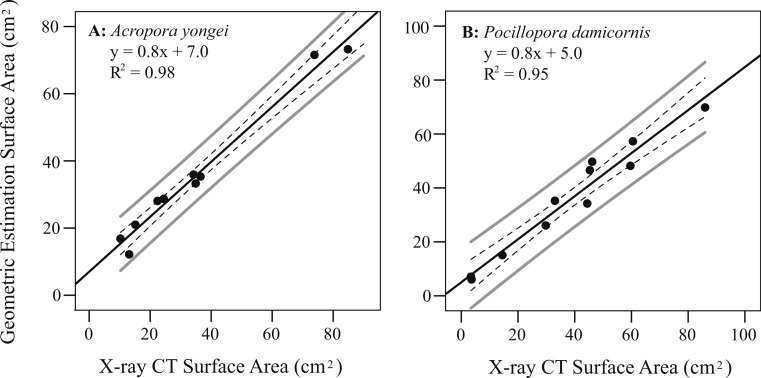
Coral surface area using geometric estimation versus X-ray CT. Regression of estimated coral surface area (geometric estimation) against accurate surface area (X-ray CT) for (A) *Acropora yongei*, and (B) *Pocillopora damicornis*; mean 95% confidence (dashed), individual 95% confidence (solid grey), and prediction (solid black) lines are shown.

**Figure 6:**
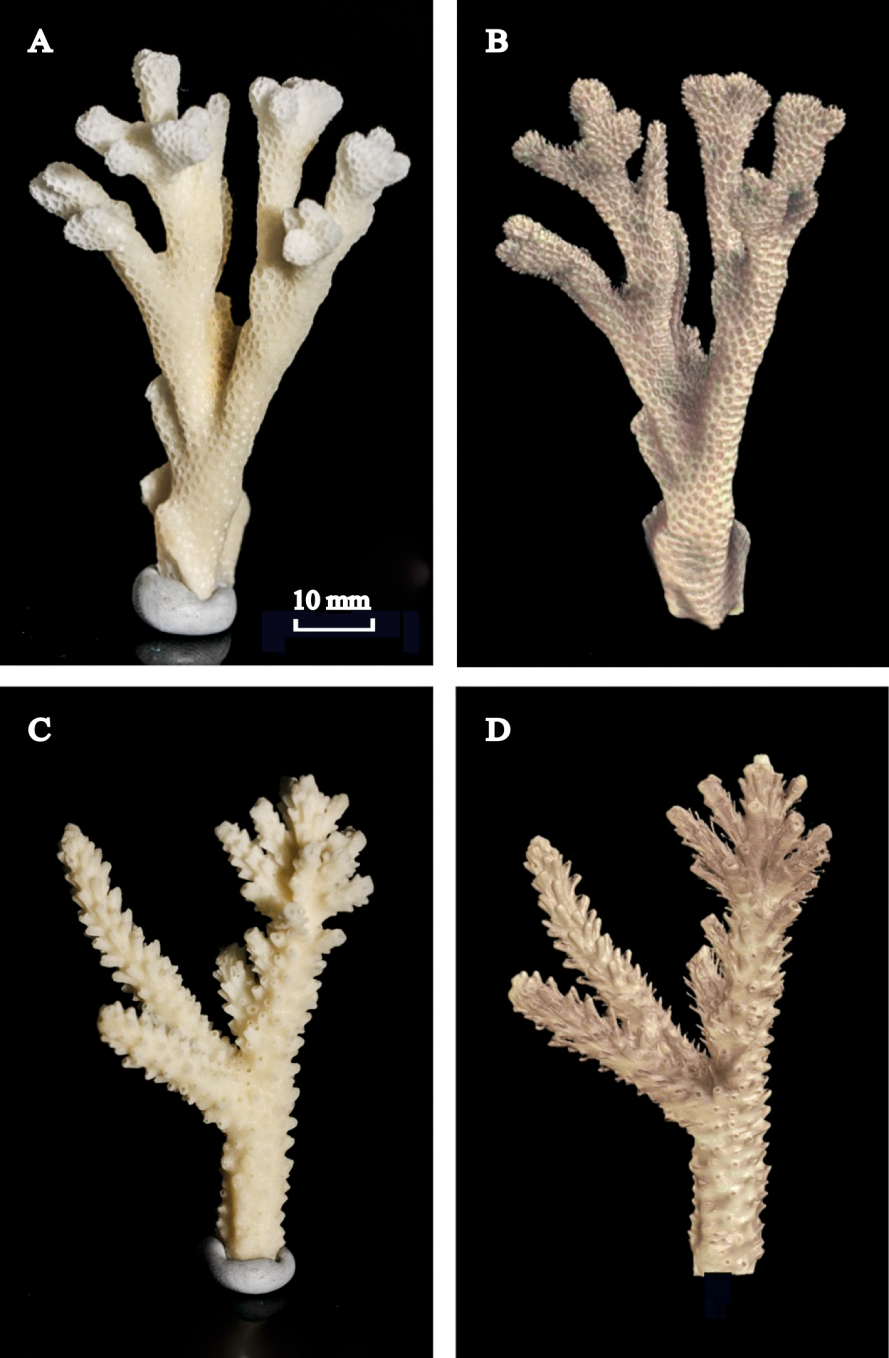
Photographed coral skeletons versus the 3D reconstructions using X-ray CT scans. Photographs of skeletons for (A) *Pocillopora damicornis* and (C) *Acropora yongei* versus (B, D) the digital reconstructions of the same branches using X-ray CT scans. Scale = 10 mm for all images. Photographs by C. Ross (2013).

**Figure 7:**
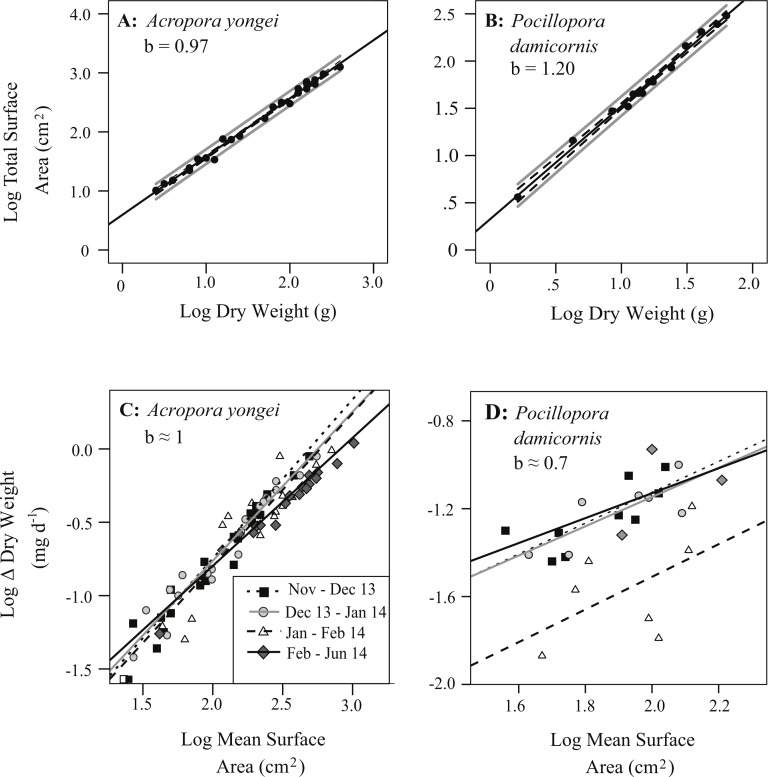
Relationships between the mass and surface area of coral colonies. Log of total colony bioactive surface area versus log of dry weight for (A) *Acropora yongei*, and (B) *Pocillopora damicornis*. The best fit linear regression of the type *y* = *mx* + *b* (black line), the 95% confidence intervals for the fitted regression (dashed) and regression predictions (solid grey) are shown. Log difference (Δ) in dry weight (mg d^−1^) versus log surface area (cm^2^) during four different growth intervals for (C) *A. yongei* and (D) *P. damicornis* at Salmon Bay, Rottnest Island.

Colony surface area estimated from geometric estimation was linearly correlated with the more accurate surface areas calculated from X-ray CT (*A. yongei*: *y* = 0.82*x* + 6.99, root mean squared error (RMSE) = 3.2 cm^2^, *R*^2^ = 0.98; *P. damicornis*: *y* = 0.8 × + 5.0, RMSE = 4.8 cm^2^, *R*^2^ = 0.95, Figs. 5A and 5B). Visual comparisons between virtual 3D reconstructions of a colony with a photograph of the same colony verified the high degree of similarity between actual fragments and the 3D reconstructions based on X-ray CT scans (Fig. 6). We further found strong linear relationships between the log of the total colony surface area and the log of dry weight for both coral species (*A. yongei*: RMSE = 1.1, *R*^2^ = 0.99, *y* = 4.1*x*^0.97^; *P. damicornis*: RMSE = 1.0, *y* = 2.1*x*^1.20^, *R*^2^ = 0.99, Figs. 7A and 7B). Rates of coral calcification were predominantly independent of size as represented by total surface area in *A. yongei* (*b* ≈ 1 ± 0.03 where Δ*M* ∝ SA^*b*^, *R*^2^ = 0.92, Fig. 7C). The relationship between calcification rate and size was, however, less clear for *P. damicornis*, which exhibited a modest allometric decrease with total surface area (*b* ≈ 0.7 ± 0.26 where Δ*M* ∝ *SA^b^*, *R*^2^ = 0.18, Fig. 7D). Thus, rates of coral calcification were largely insensitive to size; results consistent with prior studies of metabolic allometry in coral (Jokiel & Morrissey, 1986; Sebens, 1987; Elahi & Edmunds, 2007).

**Figure 8:**
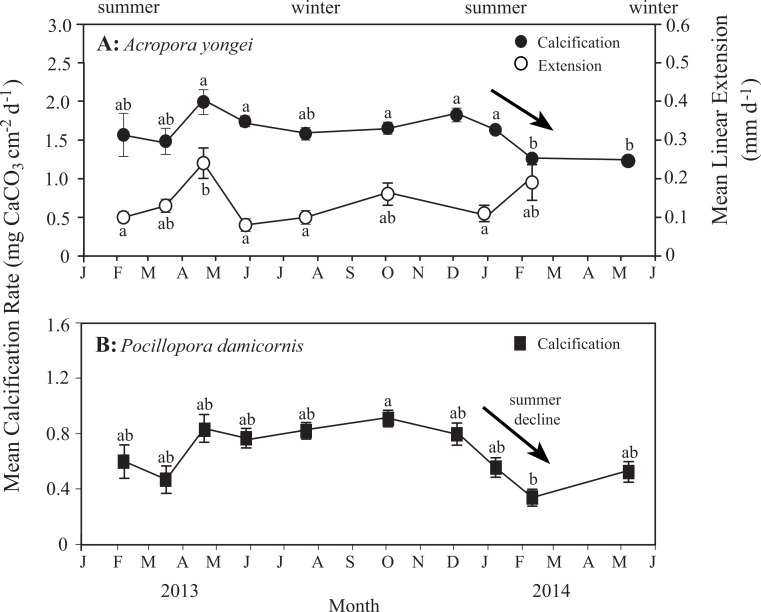
Rates of coral calcification and linear extension. (A) Mean rates of calcification (mean ± SE, black circles) with mean linear extension rates (white circles) for *Acropora yongei*, and (B) mean rates of calcification for *Pocillopora damicornis* (black squares) at Salmon Bay, Rottnest Island (letters denote groupings from Bonferroni pairwise comparisons).

Mean rates of calcification over the entire study ranged from 1.31–2.02 mg CaCO_3_ cm^−2^ d^−1^ for *A. yongei* and 0.34–0.90 mg CaCO_3_ cm^−2^ d^−1^ for *P. damicornis* (Figs. 8A and 8B). Rates of calcification were found to differ significantly for the factor of time for *A. yongei* (rANOVA, *F*_(2.8,39.6)_ = 7.0, *p* < 0.05) and *P. damicornis* (rANOVA, *F*_(3.0,17.8)_ = 3.9, *p* < 0.05). Post-hoc tests for *A. yongei* showed that the significant changes over time were attributed to the abrupt calcification rate declines of 25% during February 2014, which was followed by consistently low rates throughout autumn 2014; both time points were found to be significantly different from five other previous time points (Bonferroni post-hoc test, *p* < 0.05, Fig. 8). Similarly, post-hoc tests for *P. damicornis* showed that the significant changes over time were attributed to the abrupt calcification rate declines of 40% during February 2014, which was significantly different from one other time point (Bonferroni post-hoc test, *p* < 0.05, Fig. 8). However, following the late 2013/early 2014 summer declines, mean rates of calcification had partially recovered for *P. damicornis* (May 2014, Fig. 8). Average rates of calcification for *A. yongei* did not significantly differ between seasons (summer 2013: 1.76 ± 0.10 vs. winter 2014: 1.67 ± 0.05 vs. summer 2014: 1.60 ± 0.05 mg CaCO_3_ cm^−2^ d^−1^; mean ± SE, one-way ANOVA, *F*_(2,129)_ = 1.4, *p* > 0.05). Similarly, average rates of calcification for *P. damicornis* did not significantly differ between seasons (summer 2013: 0.64 ± 0.07 vs. winter 2014: 0.82 ± 0.05 vs. summer 2014: 0.62 ± 0.08 mg CaCO_3_ cm^−2^ d^−1^; mean ± SE, one-way ANOVA, *F*_(2,69)_ = 3.0, *p* > 0.05).

**Figure 9:**
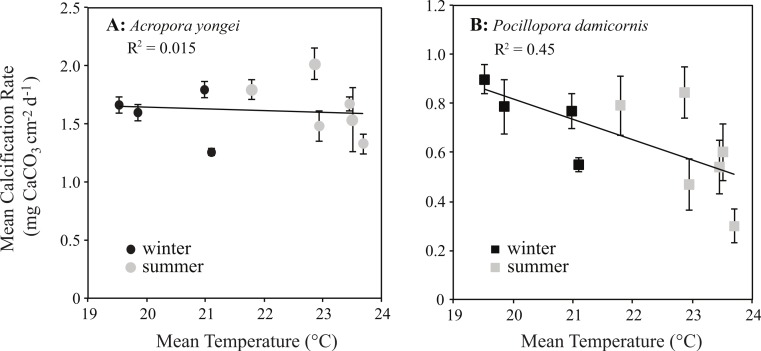
Coral calcification rates plotted against seawater temperature. Mean seasonal rates of calcification (mean ± SE) plotted against mean water temperature for (A) *Acropora yongei* (circles) and (B) *Pocillopora damicornis* (squares) in summer (grey) and winter (black) measured at Salmon Bay, Rottnest Island.

Calcification rates for *P. damicornis* showed a negative linear relationship with seasonal changes in temperature (*y* = − 0.08*x* + 2.48, RMSE = 0.15 mg CaCO_3_ cm^−2^ d^−1^, *R*^2^ = 0.45, *p* < 0.05, Fig. 9B). Below temperatures of 23 °C, however, rates of calcification for *P. damicornis* plateaued with declining temperature; thus, the relationship at lower temperatures was not significant (*b* ≈ 0 ± 0.07, *p* > 0.05, Fig. 9B). No significant correlation was found between mean rates of calcification and seasonal changes in temperature for *A. yongei* (*y* = − 0.017*x* + 2.0, *R*^2^ = 0.003, RMSE = 0.23 mg CaCO_3_ cm^−2^ d^−1^, *p* > 0.05, Fig. 9A). Similarly, no significant correlation was found between seasonal changes in daily light and calcification rates for either *A. yongei* (*R*^2^ = 0.001, *p* > 0.05) or *P. damicornis* (*R*^2^ = 0.17, *p* > 0.05).

Relatively high linear extension rates (32–74 mm y^−1^, mean of 51 ± 4 mm y^−1^, Fig. 8A) were found for *A. yongei* coral at Rottnest Island, similar to those reported more than 20 years ago (69 mm y^−1^; Marsh, 1992). Average rates of linear extension for *A. yongei* significantly differed across time (rANOVA, *F*_(7,98)_ = 4.4, *p* < 0.05); however, post hoc tests revealed that this was a result of the significantly higher rates of extension in the month of April 2013 (Bonferroni post-hoc test, *p* < 0.05), which was significantly higher than four of the other time points (i.e., February 2013, May 2013, July 2013 and January 2014). Average rates of linear extension for *A. yongei* did not significantly differ between seasons (one-way ANOVA, *F*_(2,119)_ = 1.7, *p* > 0.05). No significant correlation was found between mean rates of linear extension and rates of calcification (*R*^2^ = 0.12, *p* > 0.05).

### Shallow-water coral bleaching event

In December 2013, colonies of *P. damicornis* and *A. yongei* in Salmon Bay suffered high mortality following a bleaching event. Approximately 95% of colonies living in an extensive shallow (<0.5 m) reef flat were visibly bleached (photographs of bleaching are in Figs. S2A, S2B). However, deeper colonies (>0.5 m), including the sample coral colonies growing on tiles attached to the tripod and brick, showed no visible signs of bleaching. Subsequent monitoring within four weeks following the bleaching event showed that all of the previously bleached coral had died and were overgrown by filamentous algae.

## Discussion

This study measured coral linear extension, a method that has long been used to measure the growth of both branching and massive coral (mm y^−1^; Shinn, 1966; Oliver, Chalker & Dunlap, 1983; Lough & Barnes, 2000), combined with measurements of changes in skeletal weight normalised to the colony surface area (mg CaCO_3_ cm^−2^ d^−1^) using state-of-the-art X-ray CT scanning (Laforsch et al., 2008). Linear extension rates for *A. yongei* at Salmon Bay, Rottnest Island (32–74 mm y^−1^, mean of 51 ± 4 mm y^−1^, Fig. 8A) were found to be similar to rates reported for *Acropora* spp. from other more developed reef systems in both the tropics (63 mm yr^−1^ at the Central Great Barrier Reef and 58 mm yr^−1^ at the Maldives; Browne, 2012; Morgan & Kench, 2012) and at high latitude (33.5 mm yr^−1^ at Lord Howe Island and 37–76 mm y^−1^ at The Houtman Abrolhos Islands; Harriott, 1998; Anderson, Heron & Pratchett, 2014). However, seasonal changes in rates of calcification for branching coral at Salmon Bay did not correlate with seasonal changes in rates of extension; results that are consistent with much earlier work (Gladfelter, 1984; Brown, Syarani & Tissier, 1985). While seasonal and inter-annual variation in the growth of massive coral is expressed mainly through changes in skeletal extension (Lough & Barnes, 2000), the relationship between calcification and linear extension in branching coral is likely more complicated given their more varied and complex morphologies (Browne, 2012; Comeau et al., 2014b). Thus, although linear extension in branching species provides one measure of coral growth, these rates mainly reflect how fast the coral occupies space within the reef. In contrast, changes in total skeletal weight normalized to the living coral surface area better reflects the total amount of mass and energy directed toward biomineralization by the coral, as surface area is a major factor dictating the total amount of light, carbon, and nutrients that a coral can absorb (Vollmer & Edmunds, 2000; Laforsch et al., 2008). We therefore focused our efforts on measuring surface area-normalized rates of calcification since it is likely a better metric for comparing the effects of environmental change on rates of coral growth across different taxa and morphologies (Comeau et al., 2014b). Although there have been numerous measurements of coral growth reported in the literature, these measurements have varied widely in terms of how they both defined and calculated coral calcification rates from basic physiological metrics (e.g., % change in weight per unit time, or extension rate multiplied by density). Furthermore, the majority of area-normalized calcification rates reported to date are from massive rather than branching coral species; likely due to the difficulty or uncertainty in measuring the surface area of branching coral. Consequently, there are only a limited number of area-normalized calcification rates for branching coral with which to compare our own results.

Our rates of calcification for both *A. yongei* (1.31–2.02 mg CaCO_3_ cm^−2^ d^−1^) and *P. damicornis* (0.34–0.90 mg CaCO_3_ cm^−2^ d^−1^) at Salmon Bay, Rottnest Island were comparable to coral from other high latitude sites such as *Porites astreoides* (1.45–2.19 mg CaCO_3_ cm^−2^ d^−1^) and *Diploria strigosa* (0.68–1.78 mg CaCO_3_ cm^−2^ d^−1^) in Bermuda at 32°N (Venti, Andersson & Langdon, 2014). Calcification rates at Rottnest Island were also comparable to tropical coral species grown in mesocosms producing similar light, flow speeds, and water temperatures similar to those found in those corals’ native habitat (∼0.9–1.4 mg CaCO_3_ cm^−2^ d^−1^ for *Acropora pulchra* and ∼0.5–1.2 mg CaCO_3_ cm^−2^ d^−1^ for *P. damicornis*; Comeau et al., 2014a; Comeau et al., 2014b). However, we note that the foil method (Marsh, 1970) used by Comeau et al. (2014a) and Comeau et al. (2014b) can overestimate surface area in *Acropora* spp. by up to 60% (Veal et al., 2010) and could have caused an underestimation in the respective calcification rates for those tropical coral. Recently, Foster et al. (2014) found that *A. pulchra* within the tropical reef system at Coral Bay, Ningaloo Reef in WA (23.1°S) calcified at rates between 0.8–1.4 mg CaCO_3_ cm^−2^ d^−1^; however, they used geometric estimation to calculate surface area, which we found overestimated the true surface area by ∼20%. Thus, the rates of *A. pulchra* calcification that they reported at Ningaloo Reef are more likely to be the range of 1.0–1.7 mg CaCO_3_ cm^−2^ d^−1^; values that are nonetheless comparable to the annual calcification rates that we measured for *A. yongei* at Rottnest Island, ∼1,000 km south of Ningaloo Reef. The calcification rates for *P. damicornis* at Salmon Bay, Rottnest Island were also similar to rates measured during the same years by Foster et al. (2014) at Ningaloo Reef (0.4–0.9 mg CaCO_3_ cm^−2^ d^−1^). Foster et al. (2014) measured surface area in *P. damicornis* using the more accurate method of X-ray CT. Thus, both the summer and winter calcification rates at Salmon Bay, Rottnest Island are comparable to rates for similar coral taxa in more tropical environments despite the much lower temperatures for this high latitude environment (18.2°–24.3 °C for Rottnest vs. 23°–29 °C for Coral Bay, 27°–28 °C for Moorea; Gattuso et al., 1996; Falter et al., 2014).

The absence of any normal seasonality in the calcification rates for either *A. yongei* or *P. damicornis* at Salmon Bay, Rottnest Island stands in contrast to the majority of studies showing reduced rates of coral growth during winter months and higher rates during summer; particularly for coral living at high latitude (Crossland, 1981; Fallon et al., 1999; Howe & Marshall, 2002; Rodolfo-Metalpa et al., 2009; Venti, Andersson & Langdon, 2014). This could be due to a number of counteracting factors such as changing light, temperature, nutrients, and carbonate chemistry. While light levels at Rottnest Island were more than two and a half times higher in summer than in winter, the relatively high water clarity at Rottnest Island (Salmon Bay) indicates that coral are growing under light levels similar to those measured year-round on the shallow reef flats of Ningaloo Reef, ∼1,000 km to the north (Falter et al., 2012). Thus, our data suggest that *P. damicornis* and *A. yongei* at the more southerly location of Salmon Bay, Rottnest Island are likely obtaining sufficient light in winter, and hence, this is not limiting their growth. Similarly, variation of Ω_ar_ at Rottnest Island on seasonal time scales of 0.4–0.7 units would have only exerted a minor influence on calcification rates (∼10%; Falter et al., 2012; Venti, Andersson & Langdon, 2014), given the ability of many coral species to physiologically control the internal chemistry of the calcifying fluid (Venn et al., 2011; McCulloch et al., 2012).

Higher levels of dissolved inorganic nutrients in winter compared with summer may have helped sustain higher rates of winter coral growth than would have otherwise occurred. In winter, total dissolved inorganic nitrogen was roughly double that of summer and phosphate concentrations were more than five times as high, yet nutrient concentrations were still within ‘typical’ limits for coral reefs worldwide year-round (<1 µM; Atkinson & Falter, 2003). While very high concentrations of nutrients can actually inhibit rates of coral calcification (Ferrier-Pages et al., 2000; Silverman, Lazar & Erez, 2007), more modest increases in nutrient concentrations can enhance skeletal growth (Tanaka et al., 2007; Chauvin, Denis & Cuet, 2011). Thus, the observed modest amount of nutrient enrichment may have resulted in winter calcification rates at Salmon Bay, Rottnest Island being higher than would have occurred if nutrient concentrations remained at summer levels.

The timing of the bleaching event in mid-December 2013 raises the possibility that some form of thermal stress could have been responsible for the seasonal decline in calcification rates during the 2013 summer and 2014 autumn. While coral calcification rates generally peak close to the summer maximum temperatures, prolonged increases of 1 °C or more above this can cause bleaching and declines in growth (Jokiel & Coles, 1977). Temperatures at the depths where our experimental coral were growing (1.5 m and 2.5 m) rarely exceeded 24.5 °C for more than a few hours (Fig. 3) and bleaching was limited to the top-most ∼0.5 m of the water column. Thus, we cannot confirm that thermal stress was the main cause of the late summer decline in calcification rates without supporting measurements of the physiological state of the coral at that time (e.g., symbiont photochemical efficiency, chlorophyll *a* concentration, symbiont densities). We recognise, however that a lack of visible bleaching does not preclude the existence of thermal stress (Foster et al., 2014) and that corals can show depressed growth prior to reaching the bleaching threshold (Cantin et al., 2010).

Although the temperature-dependent growth response of tropical coral is known to vary geographically due to thermal acclimatization to a specific temperature range (Clausen & Roth, 1975), relatively little is known about the specific temperature–growth response curves for hermatypic coral at high latitude. Understanding the sensitivity of coral calcification rates to temperature could have implications for coral growth under expected future warming. Prior work has shown that the growth rates of hermatypic coral generally decline with falling temperatures below some optimal growth temperature that is 2°–3 °C below the mean monthly maximum for that environment (Clausen & Roth, 1975; Jokiel & Coles, 1977; Marshall & Clode, 2004; Howe & Marshall, 2002). In contrast, we found that the calcification rates for *P. damicornis* were surprisingly insensitive to seasonal changes in temperature below 23 °C, while calcification rates for *A. yongei* appeared to show no clear temperature–growth trend (Fig. 9). Thus, calcification rates in *A. yongei* and *P. damicornis* did not appear to conform to expected temperature–growth trends (Clausen & Roth, 1975; Jokiel & Coles, 1977; Marshall & Clode, 2004; Rodolfo-Metalpa et al., 2009). Furthermore, calcification in both species exhibited an unexpected negative sensitivity to seasonal temperatures above 23 °C; with *P. damicornis* showing marked declines in both summers and *A. yongei* showing declines in the summer of 2014 (Fig. 9). This was somewhat surprising given that branching Acroporids and Pocilloporids at Rottnest Island presumably originated from further north in the warmer Houtman Abrolhos Islands (i.e., 20°–26 °C; Foster et al., 2014) and are considered common reef-building coral in more tropical environments. That this sensitivity occurred at a temperature just ∼0.7 °C lower than the maximum monthly mean recorded temperature of 23.7 °C may indicate some kind of trade-off between robust rates of growth at lower temperatures (<20 °C) with a heightened sensitivity to even modestly warmer temperatures (>23 °C). Our findings therefore indicate that *A. yongei* and *P. damicornis* at Rottnest Island may have shifted their thermal tolerance range to support tropical rates of calcification at much cooler water temperatures within a regionally defined seasonal temperature range.

## Conclusions

The present study provides evidence of robust and perennial coral growth rates in a high latitude environment where hermatypic coral are in the early stages of colonization and expansion that could potentially lead to the development of a structurally extensive coral reef. To our knowledge, this was the first study to measure long-term in situ calcification rates of coral colonies at Rottnest Island and therefore provides baseline records for detecting future changes in calcification rates. While current conditions at Salmon Bay, Rottnest Island generally satisfy the main requirements for coral reef formation with respect to temperature, light, and nutrients (Veron, 1995), expected trends in climate-driven ocean warming suggests that the waters surrounding Rottnest Island will continue to warm (Feng et al., 2013; Zinke et al., 2014) and may therefore become even more suitable for the growth of hermatypic coral. A similar mechanism driving the poleward expansion of scleractinian coral is currently occurring along the northern islands of Japan (Yamano, Sugihara & Nomura, 2011) and on the east coast of Australia (Baird, Sommer & Madin, 2012). Nonetheless, the potential for places like Rottnest Island to act as a long-term future refuge for reef-building coral will still ultimately depend on the capacity of these coral to increase their upper thermal tolerance range and to endure the increasingly frequent episodic warming events expected to occur at high-latitude sites (Pandolfi et al., 2011; Van Hooidonk, Maynard & Planes, 2013). Rottnest Island therefore provides a unique natural laboratory in which to further study the ongoing effects of climate change on the growth and proliferation of scleractinian coral in a high latitude environment.

## Supplemental Information

10.7717/peerj.781/supp-1 Figure S1 Distribution of Acroporid coral in Salmon Bay, Rottnest Island Maps showing the distribution of *Acropora yongei* coral (1–red circle per colony) in Salmon Bay, Rottnest Island, measured using a hand-held GPS on snorkel in December 2013.

10.7717/peerj.781/supp-2 Figure S2 Bleached coral on the shallow reef Photographs showing (A) bleached *Acropora yongei*, and (B) bleached *Pocillopora damicornis* on the shallow reef at Rottnest Island in December 2013.

10.7717/peerj.781/supp-3 Figure S3 Diurnal variations of seawater pH_T_ Continuous measurements of pH_T_ in Salmon Bay, Rottnest Island taken during (A) the 19th–25th of January 2014 and (B) the 18th–30th of July 2014. The solid red lines denote the diurnal averages of 8.03 for January and 8.07 for July. The dashed black lines represent the hypothetical pH in equilibrium with the atmosphere of 8.044 for January and ∼8.048 for July at in situ temperatures (*p*CO_2_ = 395 µatm).

## Additional Information and Declarations

### Field Study Permissions

The following information was supplied relating to field study approvals (i.e., approving body and any reference numbers): The Rottnest Island Authority (RIA) provided approval and permits for this research (#2012/164683 and 2014/227143). The Government of Western Australia Department of Parks and Wildlife (DPaW) provided research permits and license to take fauna for scientific purposes (#SF009096 and #SF009705) and the Government of Western Australia Department of Fisheries provided research permits and exemption from the Fish Resources Management Act 1994 (#2202 and #2410).

### Data Deposition

The following information was supplied regarding the deposition of related data: DOI 10.5061/dryad.tv179.
