# Supplementary figures and images for: Perennial growth of hermatypic corals at Rottnest Island, Western Australia (32°S)
Source: PeerJ. 2015 Feb 24;3:e781. doi: 10.7717/peerj.781 (PMC4349054; doi:10.7717/peerj.781)

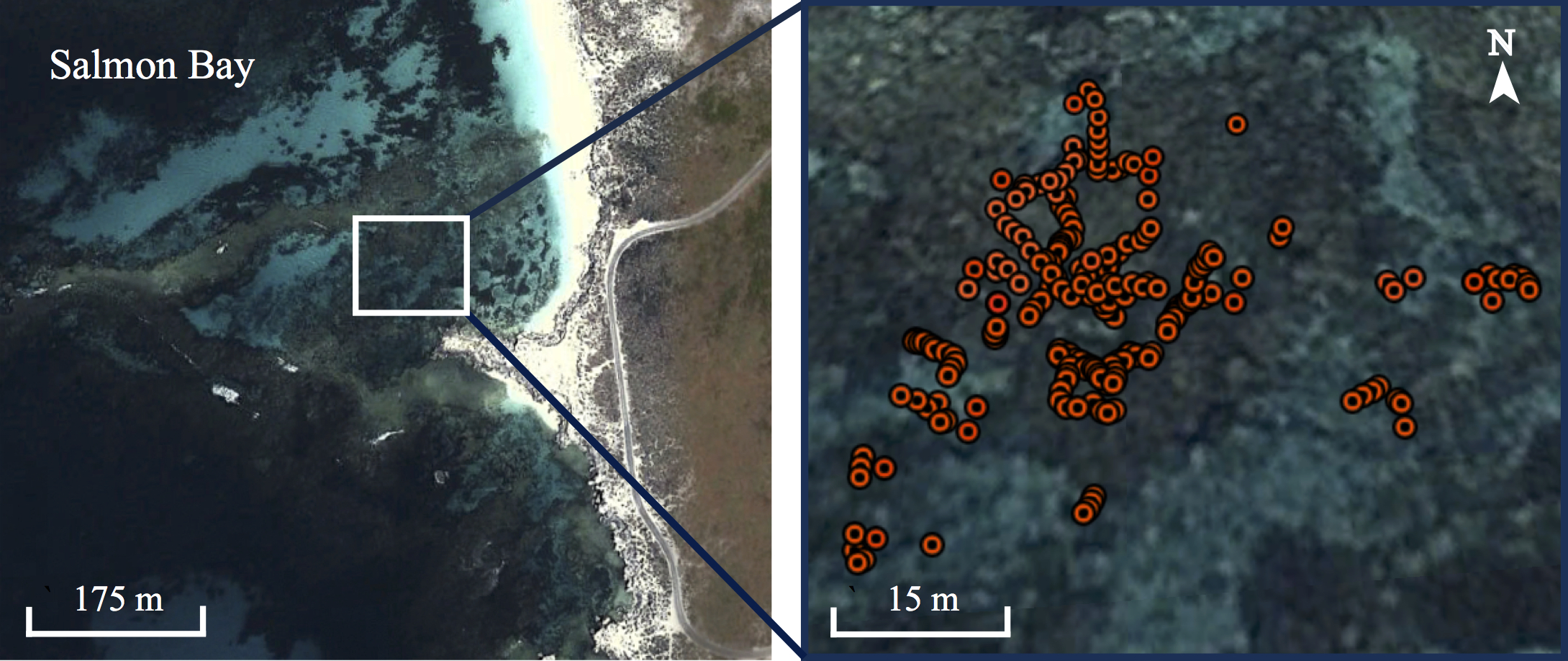

Supplement: Figure S1 — Maps showing the distribution of Acropora yongei coral (1–red circle per colony) in Salmon Bay, Rottnest Island, measured using a hand-held GPS on snorkel in December 2013. [file peerj-03-781-s001.png]

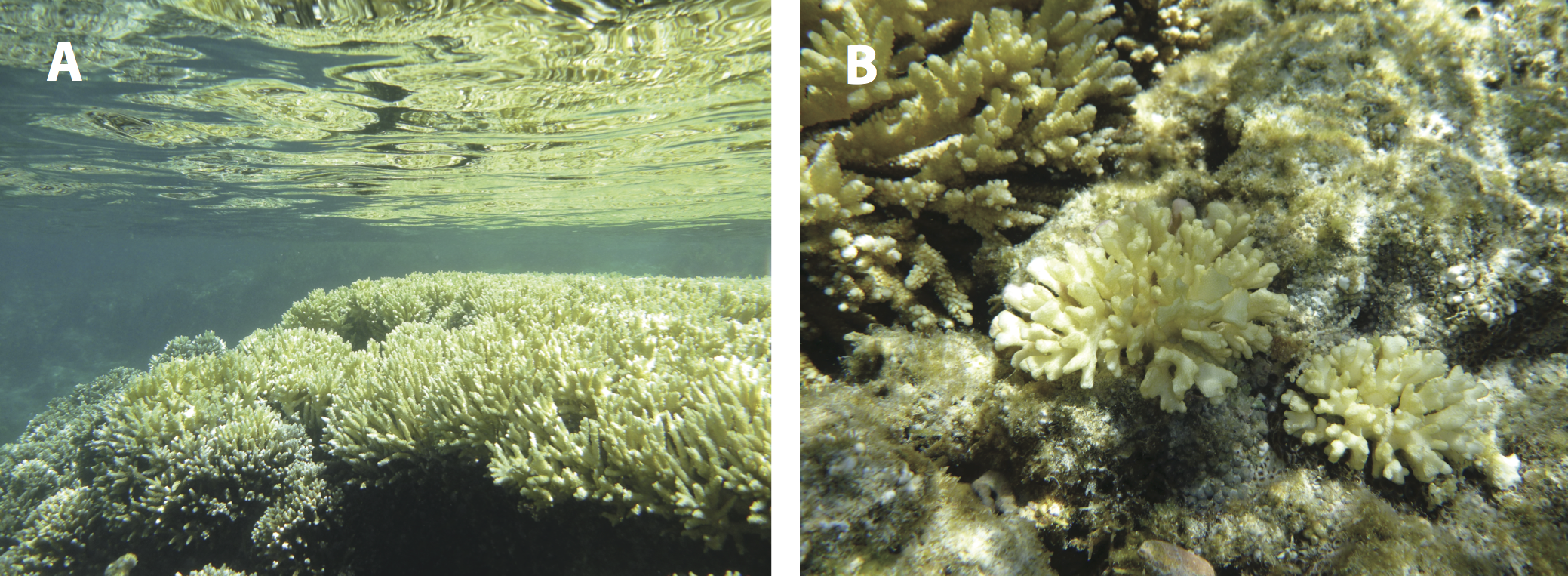

Supplement: Figure S2 — Photographs showing (A) bleached Acropora yongei, and (B) bleached Pocillopora damicornis on the shallow reef at Rottnest Island in December 2013. [file peerj-03-781-s002.png]
